# The outcome of treatment limitation discussions in newborns with brain injury

**DOI:** 10.1136/archdischild-2014-307399

**Published:** 2014-12-04

**Authors:** Marcus Brecht, Dominic J C Wilkinson

**Affiliations:** 1 Women's and Children's Hospital, Adelaide, Australia; 2 Flinders Medical Centre, Adelaide, Australia; 3 Faculty of Philosophy, Oxford Uehiro Centre for Practical Ethics, University of Oxford, Oxford, UK; 4 Robinson Institute, Discipline of Obstetrics and Gynaecology, University of Adelaide, Adelaide, Australia; 5 John Radcliffe Hospital, Oxford, UK

**Keywords:** Ethics, Neonatology, Palliative Care

## Abstract

**Background:**

Most deaths in severely brain-injured newborns in neonatal intensive care units (NICUs) follow discussions and explicit decisions to limit life-sustaining treatment. There is little published information on such discussions.

**Objective:**

To describe the prevalence, nature and outcome of treatment limitation discussions (TLDs) in critically ill newborns with severe brain injury.

**Design:**

A retrospective statewide cohort study.

**Setting:**

Two tertiary NICUs in South Australia.

**Patients:**

Ventilated newborns with severe hypoxic ischaemic encephalopathy and periventricular/intraventricular haemorrhage (P/IVH) admitted over a 6-year period from 2001 to 2006.

**Main outcome measures:**

Short-term outcome (until hospital discharge) including presence and content of TLDs, early childhood mortality, school-age functional outcome.

**Results:**

We identified 145 infants with severe brain injury; 78/145 (54%) infants had documented TLDs. Discussions were more common in infants with severe P/IVH or hypoxic–ischaemic encephalopathy (p<0.01). Fifty-six infants (39%) died prior to discharge, all following treatment limitation. The majority of deaths (41/56; 73%) occurred in physiologically stable infants. Of 78 infants with at least one documented TLD, 22 (28%) survived to discharge, most in the setting of explicit or inferred decisions to continue treatment. Half of long-term survivors after TLD (8/16, 50%) were severely impaired at follow-up. However, two-thirds of surviving infants with TLD in the setting of unilateral P/IVH had mild or no disability.

**Conclusions:**

Some critically ill newborn infants with brain injury survive following TLDs between their parents and physicians. Outcome in this group of infants provides valuable information about the integrity of prognostication in NICU, and should be incorporated into counselling.

**What is already known on this topic**

- Most deaths in newborn intensive care follow decisions by parents and doctors to limit life-sustaining treatment.
- Some newborn infants who die in neonatal intensive care unit are physiologically stable and have life-sustaining treatment withdrawn or withheld because of concerns about future quality of life.

**What this study adds**

- A high proportion of critically ill newborns with severe brain injury had documented treatment limitation discussions between parents and doctors.
- Almost one-third of these newborns ultimately survived to discharge from hospital.
- A majority of infants who had treatment limitation discussions either died or were severely disabled; however, some infants (particularly following intraventricular haemorrhage) were not impaired.

## Introduction

Despite improvements in the care of sick newborns, some infants admitted to neonatal intensive care units (NICUs) die. In North America, Australia and Northern Europe, the majority of deaths in NICUs follow decisions to limit life-sustaining treatment.^1–5^ In these units, it is rare for infants to die on the ventilator, or while receiving cardiopulmonary resuscitation (CPR).^2^ ^5^ Some decisions to limit treatment are taken for infants who are physiologically unstable and likely to die soon regardless of decisions.^6^ However, up to 50% of treatment limitation decisions (TLDs) in NICUs occur in stable infants, on the basis of predicted poor prognosis and reduced quality of life.^2^ ^4^ ^5^ The majority of the latter decisions occur in newborn infants with brain injury.^2^

Previous studies of TLDs in NICUs have focused on infants who died.^1–5^ Yet, anecdotal reports suggest that some infants who have treatment limited, nevertheless, survive.^7^ Moreover, some parents, when asked about limitation of treatment, request that support continue.^8^ ^9^ There is little information available on how often TLDs occur in the NICU.^10^ ^11^ It is unclear how often newborns survive following TLDs.

The aim of this study was to describe the prevalence and nature of TLDs in a cohort of critically ill newborns with severe brain injury and to assess the outcomes of these discussions. In addition, we sought to describe the school-age functional outcome in survivors. This group of infants may shed important light on the accuracy of prognostication for critically ill infants.^12^

## Patients and methods

In South Australia, there are two tertiary NICUs servicing a population of approximately 1.6 million, with 18 000 births per year. We searched the neonatal databases at both the NICUs to identify newborns admitted between 1 January 2001 and 31 December 2006 with a clinical diagnosis of moderate or severe periventricular/intraventricular haemorrhage (P/IVH) or hypoxic–ischaemic encephalopathy (HIE). Moderate-to-severe IVH was defined as a radiological diagnosis of grade III/IV P/IVH.^13^ Moderate-to-severe HIE was defined as a clinical diagnosis by the treating neonatologist of Sarnat stage 2 or 3 encephalopathy.^14^

We excluded infants who had received <4 h of mechanical ventilation as TLD were less likely to have occurred. Medical records were reviewed for all eligible infants by one of the authors (MB).

TLDs were recorded where there was clear evidence from medical or nursing notes that palliative care or limitation of treatment had been discussed with parents. Discussions about prognosis alone were excluded, as were discussions occurring outside the NICU.

We recorded options that had been presented to parents. Infants were classified according to their physiological stability and need for respiratory support at the time of discussions (table 1).^2^ The conclusions of each discussion (where documented) were recorded, as well as specific elements of any treatment limitation. In-hospital outcome was recorded. Deaths were classified into four categories.^2^

**Table 1:** Physiological stability classification

1. Critically unstable/moribund	Deteriorating despite high levels of intervention and full organ support. Infants were included in this category if they had protracted bradycardia or anuria for >24 h, hypotension despite volume infusion and inotropes; persistent desaturation despite mechanical ventilation and 100% oxygen.
2. Stable, requiring high level of support	Infants requiring a high level of organ support, but not meeting criteria 1 (above). Infants were included in this category if they were mechanically ventilated requiring ≥80% oxygen and/or a mean airway pressure of ≥14 cm H_2_O; had vasopressor-resistant hypotension requiring infusion of ≥20 µg/kg/min dobutamine/dopamine or requiring adrenaline infusion.
3. Physiologically stable	All other infants not fulfilling the above criteria

Criteria were modified from Verhagen *et al*,^2^ with addition of an additional category (2) to distinguish decisions likely to be based on quality of life, from decisions that may reflect a high (but not inevitable) chance of death.

Long-term functional outcome was assessed from the most recent documented follow-up. Functional outcome and need for support with activities of daily life were assessed from outpatient or inpatient encounters, clinic letters and formal developmental assessments and classified according to the modified Glasgow Outcome Scale (GOS).^15^ Children with cerebral palsy were classified according to the gross motor function classification system as assessed by a paediatrician.^16^

Fisher's exact test was used to compare the frequency of TLDs in different diagnostic groups, and the frequency of outcome between different groups of infants (SAS software, SAS Institute, Cary, North Carolina, USA). Ethics committee approval for the study was obtained from both the hospitals.

## Results

**Figure 1:**
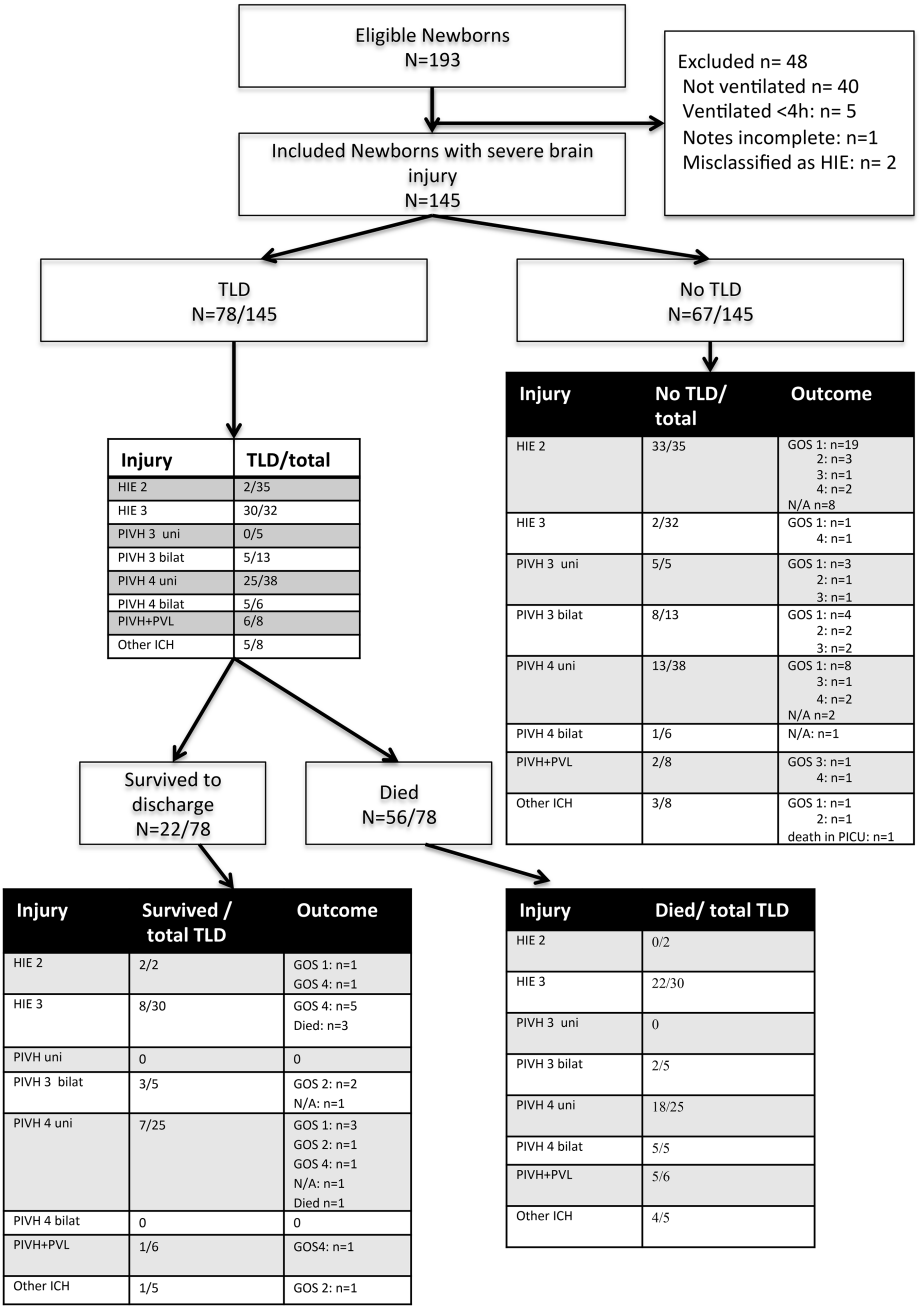
Flowchart of cohort, including outcome at latest follow-up (GOS, Modified Glasgow Outcome Score); N/A—long-term outcome data not available; TLD—treatment limitation discussion; HIE, hypoxic–ischaemic encephalopathy; P/IVH, periventricular/intraventricular haemorrhage; PVL, periventricular leucomalacia; uni, unilateral; bilat, bilateral; ICH, intracranial haemorrhage. Modified GOS categories: 1—functionally normal, 2—mildly disabled but likely independent, 3—moderately disabled and dependent on care, 4—severely disabled and totally dependent on care.^15^

During the study period, 3153 newborns were admitted to the two NICUs. We identified 193 infants with severe brain injury. Forty-eight were excluded, most on the basis of not requiring respiratory support (figure 1). (None of the excluded infants died prior to discharge. Most had moderate HIE.)

We analysed 145 cases, including 67 infants with HIE and 62 with P/IVH (see online supplementary appendix table 1 for demographic characteristics). Eight infants had P/IVH combined with evidence of periventricular leucomalacia (PVL). A further eight infants had intra-cerebral haemorrhage different from the typical P/IVH pattern in pre-terms.

TLDs were identified in 78/145 (54%) infants. Discussions were more common in infants with stage 3 HIE (compared with stage 2, p<0.0001), and in infants with grade IV P/IVH (compared with grade III, p<0.01) (table 2). One to six TLDs were documented per infant (total of 176 discussions (see online supplementary appendix figure 1)). Forty-seven infants (47/78, 60%) had more than one TLD. In infants with HIE, the first TLD occurred on the day of birth in 14/32 (43%).

**Table 2:** Diagnosis and outcome in infants with TLD compared with infants without TLD

	TLD	No TLD
Diagnosis		
HIE—stage 2	2 (5.7%)	33 (94.3%)
HIE—stage 3	30 (93.8%)*	2 (6.2%)*
P/IVH—grade III	5 (27.8%)	13 (72.2%)
P/IVH—grade IV	30 (68.2%)*	14 (31.8%)*
Outcome		
Survived	18 (23.1%)†	66 (98.5%)†
GOS 1	4 (22.2%)†	36 (54.5%)†
GOS 2	4 (22.2%)	7 (10.6%)
GOS 3	0	6 (9.1%)
GOS 4	8 (44.4%)†	6 (9.1%)†
N/A	2 (11.1%)	11 (16.7%)
Died‡	60 (76.9%)†	1 (1.5%)†

For diagnosis, percentages are expressed as a proportion of each category with or without TLD.

For neurological outcome, percentages are expressed as a proportion of surviving infants within each outcome category. Survival/Deaths are expressed as a proportion of all infants with/without TLD.

*p<0.05 (Fisher's exact test) (comparing frequency of treatment limitation decisions between infants with stage 3 versus stage 2 HIE, and grade IV versus grade III P/IVH).

†p<0.05 (Fisher's exact test) (comparing frequency of outcome between infants with/without TLD).

‡Death includes in-hospital and post-discharge deaths.

GOS, Glasgow Outcome Scale; HIE, hypoxic–ischaemic encephalopathy; P/IVH, peri-/intraventricular haemorrhage; TLD, treatment limitation discussion.

**Figure 2:**
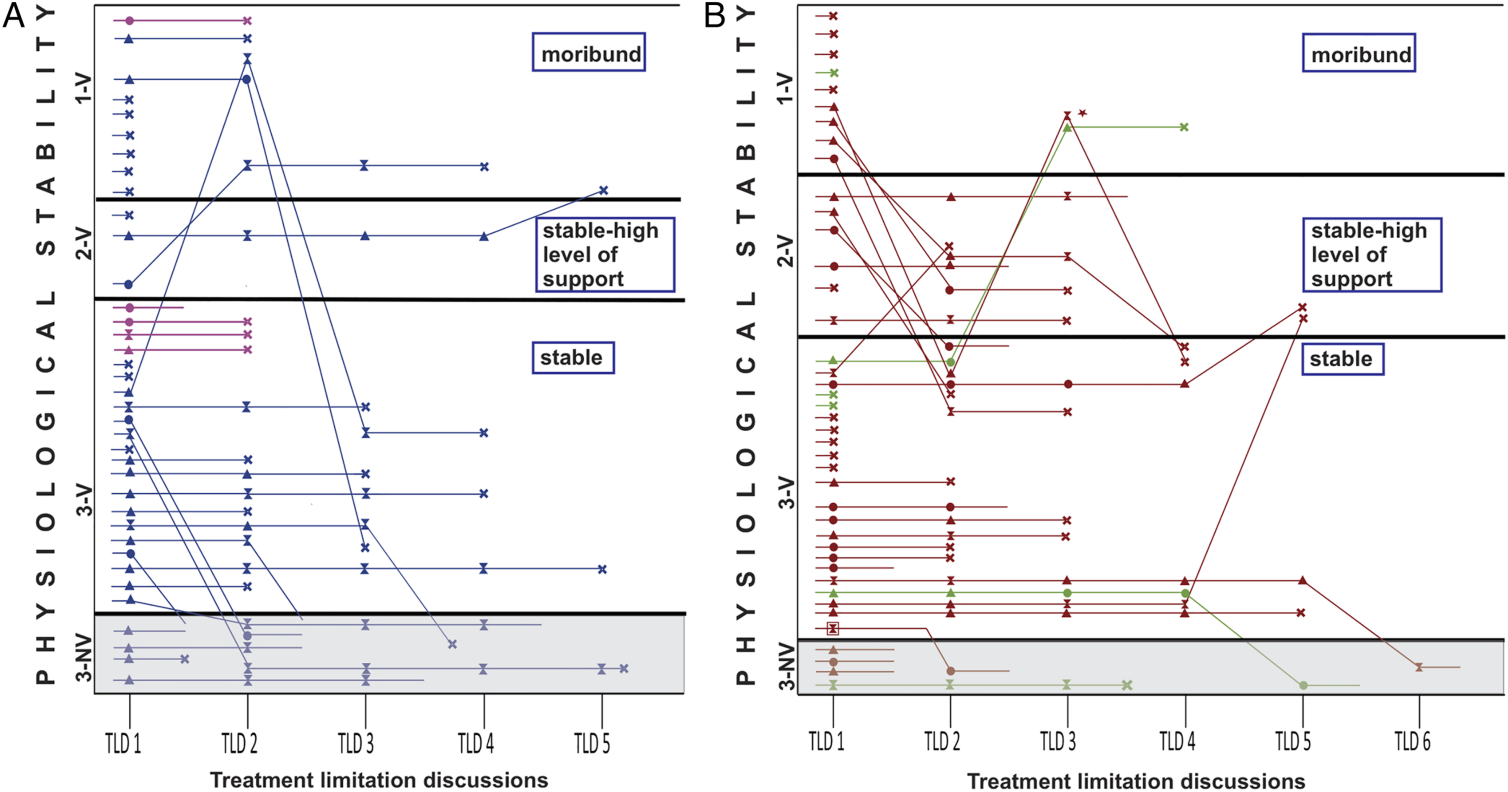
Severity of illness and treatment limitation discussions (TLD). Each line represents the course of an individual infant. Decisions are classified according to the infant's physiological stability at the time of discussion, while the symbols represent the result of discussions. The shaded area in the lower figure indicates infants who were not ventilated (NV) at the time of discussion. (A) Newborns with hypoxic–ischaemic encephalopathy and other intracranial pathology (‘other intra-cerebral haemorrhage’); (B) newborns with P/IVH (periventricular/intraventricular haemorrhage) and P/IVH+ periventricular leucomalacia. ▲, survived-no parental decision documented; X, died following decision to limit or withdraw treatment; 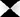, limitation/withdrawal -survived; ● survived-parental decision to continue treatment; 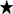, unilateral decision; open-ended lines, patient survived to discharge; □, decision made at referring hospital.

The majority of the discussions took place when newborns were physiologically stable (124/176, 70.5%), while a small proportion were moribund at the time (29/176, 16.4%, figure 2, see also the online supplementary appendix figure 2). In 28 TLDs, the parental decision was to continue intensive care; in 99 TLDs, some form of limitation was documented. In 49 TLDs, it was unclear whether the parents had made a decision; in all but one case intensive care was continued; this newborn died at 9 hours following severe HIE and was moribund. In one case of a critically unstable pre-term infant with grade 2 and 4 P/IVH, a unilateral decision was made by the treating team not to perform CPR in case of an adverse event; this baby later died following a TLD with parental agreement.

Mechanical ventilation was the treatment most frequently withdrawn. Specific treatments that were to be withheld included reintubation (16 cases), continuous positive airway pressure (1), CPR (28) and other therapies (15). In 11 documented discussions, details of treatment to be limited were not provided.

In eight cases, some form of limitation involving feeding was discussed. In one case with HIE, withholding nasogastric tube (NGT) feeds was agreed; however, this was later revoked on the parents’ request. In two cases, parents declined withholding of NGT feeds. For a fourth infant with HIE, it was initially agreed to only offer oral feeds; this was later changed to NGT feeds at a reduced volume due to frequent regurgitation. The infant died on day 18. In a fifth case, parents were offered withdrawing NGT feeds, agreed, and the infant survived on oral feeds alone (the infant was unimpaired at age 3). In the one remaining HIE case, no parental decision was documented: the infant died later at day 50. In a case with congenital hydrocephalus and IVH, agreed withholding of NGT feeds was not implemented as the infant died following extubation. In a case with IVH and PVL, an NGT was inserted for administration of daily phenobarbitone, while feeds were being withheld. In this case subcutaneous hydration was also withheld. The infant died 10 days after the documented decision to withhold fluids and feeds.

There was a high observed mortality rate in this cohort. Of all 145 cases, 56 (39%) infants died before discharge, all following one or more TLD. None of the newborns died during CPR. All newborns died with one or both parents present. One premature newborn died while connected to the ventilator in his parents’ arms. In 41 cases, deaths followed withholding/withdrawing treatment in stable newborns (see online supplementary appendix table 2).

Most deaths (31/56, 55%) occurred within the first week of life, a further 14/56 (25%) newborns died in the second week of life. Four infants died outside the NICU, three babies with HIE stage 3 died at home on day 18, 50 and 70 and one infant with progressive hydrocephalus and PVL died in the parents’ accommodation on hospital premises (day 63 of life). Two infants were re-admitted to paediatric intensive care unit (PICU) and died following a TLD later. One child died in mid-childhood.

Neurodevelopmental outcome was significantly better for infants without TLDs; most were functionally normal at follow-up (36/66, 54.5% table 2). Of the 78 infants who had TLDs, 22 (28%) survived to discharge. Eight of 22 (32%) parents had decided in favour of continued treatment, in 6/22 (27%) no clear decision was documented, while in 8/22 (32%) parents had agreed to limitation of treatment (see online supplementary appendix table 3). Follow-up data were available for 20/22 (figure 1). Eight children had severe non-ambulant cerebral palsy (GOS 4), while eight had mild or no disability (GOS 1 or 2). Four children died in infancy or early childhood. Outcome was significantly worse for infants with HIE compared with those with P/IVH (table 3). All the infants who had TLD in the context of severe HIE died or were severely disabled at follow-up. Four out of seven (57%) newborns with unilateral severe P/IVH had normal functional status or only mild disability at follow-up (the online supplementary appendix table 5 provides details of decisions along with functional outcome and age of last assessment).

**Table 3:** The influence of diagnosis on outcome in infants with TLD

	HIE (n=32)	P/IVH (n=35)		P/IVH+PVL (n=6)	Other ICH (n=5)
*Outcome*					
Died in hospital	22 (68.8%)	25 (71.4%)		4 (83.3%)	4 (80%)
Survived to discharge	10 (31.2%)	10 (28.6%)		1 (16.7%)	1 (20%)
GOS 1	1 (10%)	3 (30%)		0	0
GOS 2	0	3 (30%)		0	1 (100%)
GOS 3	0	0		0	0
GOS 4	6 (60%)	1 (10%)*	]†	1 (50%)	0
Died in infancy/childhood	3 (30%)	1 (10%)		1 (50%)	0
N/A	0	2 (20%)		0	0

Neurological outcome and death post-discharge expressed as proportion of infants surviving to discharge.

*p=0.06 (Fisher's exact test, comparing severe disability between infants with HIE and those with P/IVH).

†p<0.01 (Fisher's exact test, comparing combined outcome of death (post-discharge) or severe disability between infants with HIE and those with P/IVH).

GOS, Glasgow Outcome Scale; HIE, hypoxic–ischaemic encephalopathy; P/IVH, periventricular/intraventricular haemorrhage; PVL, periventricular leucomalacia; ICH, intra-cerebral haemorrhage; TLD, treatment limitation discussion.

## Discussion

In this retrospective study, we identified a high prevalence of TLDs in a cohort of critically ill newborn infants with severe brain injury. All deaths followed decisions to limit or withdraw treatment. However, about one-quarter of infants whose parents had held documented TLDs survived. In the majority of these cases, there was an explicit or inferred decision to continue life-sustaining treatment. Most infants surviving after TLDs were severely impaired long term.

### Treatment limitation—prevalence

Previous studies have found similarly high rates of treatment limitation (69%–94%) among infants dying in NICUs in North America and Northern Europe,^1^ ^2^ ^5^ ^17^ though lower rates have been observed (45%–54%) in Israel^18^ and South America.^19^ Prior studies included a range of underlying pathologies. A variable proportion of deaths (0%–61%) occurred in stable infants having treatment withdrawn on the basis of concerns for quality of life.^2^ ^5^ ^17^ In our study, focusing on infants with brain injury, about three-fourths of deaths fell into this ‘stable’ category.

### Survival after TLDs

In adult intensive care, a very small proportion of patients survive after treatment is withdrawn.^20–22^ A French study in paediatric intensive care described survival to hospital discharge in nine out of 30 children who had treatment limitations following a family conference.^23^

Two previous studies have examined parental treatment decisions and outcome in the NICU. The first, published in 1986, described 75 cases.^10^ TLDs occurred in about two-thirds; in four cases, parents requested continuation of intensive care; half of these babies died on the ventilator. No infant survived after limitation of treatment.

In a German study, TLDs were studied prospectively in a tertiary neonatal unit.^11^ Thirty-two infants had an agreed limitation of treatment; of these, three survived to hospital discharge, two dying at home and one surviving for more than 1 year. Three out of four infants having decisions to continue treatment were discharged alive. Neither study reported long-term outcome in survivors.

### Functional outcome after TLDs

In our study, all of the infants with documented TLDs in the setting of severe HIE were severely disabled with non-ambulant cerebral palsy at follow-up. This is consistent with earlier studies that reported 100% rates of severe disability in surviving infants with Sarnat 3 HIE.^24^ ^25^ In contrast, in recent cooling trials in HIE, 36% of infants with severe encephalopathy survived without severe impairment.^26^ This lower rate may reflect the early staging of encephalopathy in the cooling trials or possibly the influence of cooling itself. Although the cohort described here preceded the introduction of hypothermia into routine care for HIE, the majority of surviving infants (6/10) had been cooled (see online supplementary appendix table 4).

Outcome was more variable in newborn infants with TLDs in the setting of P/IVH. In previous series, unilateral parenchymal haemorrhage has been associated with cognitive abilities close to the general population and mild or no motor problems in a significant proportion of infants.^27–29^ Newborns with bilateral parenchymal lesions have been reported to have a high rate of severe impairment.^27^ In our study, no newborns with bilateral grade IV P/IVH survived.

In a study from Chicago, 100% of premature infants with abnormal ultrasound, whose caregivers believed that death was likely prior to discharge, either died or were severely impaired.^30^ The presence of a TLD might be thought to correlate with physician intuitions of demise. However, in our study, surviving infants with P/IVH and TLDs had a relatively low rate of severe impairment.

### Absence of end-of-life discussions

There was a small number of infants in this study with particularly severe brain injury but no documentation of TLDs. This included one infant with bilateral severe IVH and two infants with Sarnat stage 3 HIE, one of whom survived with significant disability. In the group of infants with unilateral grade IV P/IVH, one-third of infants did not have documented TLDs.

It was unclear why discussions did not occur in these cases. One possibility is that neonatologists took into account other features predicting a better outcome.^31^ It is possible that variability in physicians' propensity to make end-of-life decisions contributed to a lack of discussion.^32^ Finally, it is conceivable that discussions did actually take place, but were not documented.

### Ethical implications

There are a number of potential ethical implications of this study. Some newborn infants who have life-sustaining treatment withdrawn or withheld survive to discharge from hospital.^7^ From our study, and consistent with expectation, this was most likely to occur in physiologically stable infants having treatment limited on the basis of quality of life. It is important for clinicians to be cautious when predicting demise and to be aware of the possible need for ongoing palliative care.

Our cohort illustrates some of the challenging questions in the care of severely brain-injured newborns who are not dependent on respiratory support. Withdrawal of artificial nutrition and hydration has been defended as a palliative option in newborn infants,^33–35^ yet it has been reported only rarely.^36–38^ In our study, it was discussed with the parents of eight infants, including six of the survivors. In two cases, NGT feeds were withheld, but subsequently reinstated. In another, the infant was able to maintain enough oral intake to survive long term. The psychological burden on parents and staff when feeds are withheld can be substantial.^39^ ^40^

The long-term outcome of children whose parents have been offered the option of treatment limitation provides an invaluable insight into prognostication and decision making in intensive care.^12^ Although the outcome for such infants may not be equivalent to those infants who succumbed, it can identify the best achievable outcome if treatment is continued, information important for counselling. Our data support the appropriateness of decisions to potentially limit life-sustaining treatment in a cohort of term infants with severe HIE who did not receive therapeutic hypothermia. The degree of impairment in all surviving infants was sufficiently severe that intensive treatment could be judged, in retrospect, to have been ethically optional.^41^ ^42^ However, the much more variable outcome in infants with P/IVH raises questions about the justification of treatment limitation in this cohort.^43^ Two-thirds of surviving infants with unilateral severe P/IVH, whose parents had been offered treatment limitation, were minimally impaired or unimpaired at follow-up. Decisions to limit treatment on the basis of intraventricular haemorrhage need to be taken cautiously, combine ultrasound variables^31^ with clinician's experience^30^ and include counselling about uncertain prognosis.

### Limitations

This was a retrospective study and, as such, some of the findings should be treated with caution. Follow-up data were unavailable for some infants. Severely affected children are more likely to require tertiary hospital care and may be over-represented. TLDs are likely to have been richer and more nuanced than was reflected in the medical record and some discussions may not have been documented or may have been missed in infants excluded because of lack of respiratory support. Assessment of outcome for children was limited to data available from notes, and represents an incomplete picture of their function and quality of life. Finally, it is possible that the timing or content of end-of-life discussions or the outcome for infants have changed since the period of this study. Therapeutic hypothermia is now standard of care for infants with HIE, and there have been concerns that this might lead to delays in TLDs or to increased uncertainty about prognosis.^44^ ^45^ While there was no apparent difference in outcome between cooled and non-cooled surviving infants in this study, the numbers are small, and it would be useful to repeat the study in a larger sample of cooled infants.

## Conclusion

TLDs occur commonly in the NICU for infants with severe brain injury. Some newborn infants survive because of parental decisions to continue treatment, or despite explicit decisions to withhold life-prolonging treatment. Many, though not all, of these surviving children have severe life-long disability. Prospective studies are needed that combine careful documentation of the rationale for decisions with long-term follow-up. There is also a need for research into the long-term care needs of this highly vulnerable group of children and families.

## Supplementary Material

Web supplement
