# Supplementary material for: The outcome of treatment limitation discussions in newborns with brain injury
Source: Arch Dis Child Fetal Neonatal Ed. 2014 Dec 4;100(2):F155–60. doi: 10.1136/archdischild-2014-307399 (PMC4345812; doi:10.1136/archdischild-2014-307399)

APPENDIX (ONLINE ONLY MATERIAL)

Appendix Table 1: Demographics and patient characteristics

Appendix Table 2: In-hospital deaths and physiological stability at time of last TLD.

Appendix Table 3: Diagnosis and outcome in infants surviving to discharge whose parents agreed to treatment limitation, compared with infants whose parents did not agree to limitation of treatment

Appendix Table 4: Diagnosis and outcome in infants with HIE surviving to discharge who received or did not receive therapeutic hypothermia

Appendix Table 5: Outcome of survivors following Treatment Limitation Discussions

Appendix Figure 1: Distribution of number of TLDs in relation to number of newborns

Appendix Figure 2: Stability at the time of treatment limitation discussion

Appendix Table 1: Demographics and patient characteristics

1a HIE 2 (n=35)

gestational age (median (range in weeks $^{+days}$)	40^{+0} (35^{+0} - 41^{+5})
Birth weight (g; mean and range)	3392 (2120-4475)
male (n (%))	16 (45%)
outborn (n (%))	16 (45.7%)
Apgar 1 min (median [IQR])	2 [1; 3]
Apgar 5 min (median [IQR])	4 [3; 6]
Adrenaline for resuscitation (%)	16 (45%)
Cord or first postnatal pH (+/- SD)	7.10 +/-0.21
worst base deficit in mmol/l (mean +/- SD)	13.8 (+/-7.5)
Hypothermia (n (%))	24 (68.6%)

1b HIE 3 (n=32)

gestational age (median (range in weeks $^{+days}$)	39^{+0} (31^{+0} – 41^{+5})
Birth weight in g (mean and range)	3168 (1390-4320)
Male (in %)	16; (50%)
Outborn (in %)	18 (56.2%)
Apgar 1 min (median [IQR])	1 [0; 2]
Apgar 5 min (median [IQR])	2 [1;4]
Adrenaline for resuscitation (n; (%)	18 (56.2%)
cord or Initial pH (mean+/-SD)	6.89 +/- 0.31
worst base deficit in mmol/l (mean +/-SD)	19.9 (+/-7.40)
Hypothermia (n; (%))	14 (43.7%)

1c P/IVH (all grades, n=62)

Gestational age (median (range in weeks $^{+days}$)	25^{+6} (22^{+5} – 35^{+0})
Birth weight in g (mean and range)	910 (460-3480)
Male (n (%))	30 (48.4%)
multiple pregnancy (n; (%))	16 (25.8%)

Outborn (n; (%))	6 (9.7%)
Apgar 1 min (median [IQR])	4 [1;6]
Apgar5 min (median [IQR])	7 [6; 8]
antenatal steroids (n; (%))	38 (61.3%)

1d. P/IVH+PVL (n=8)

Gestational age (median (range in weeks $^{+days}$)	27^{+5} (23^{+5} - 34^{+0})
Birth weight in g (mean and range)	1325 (560-3565)
Male (n; (%))	5 (62.5)
multiple pregnancy (n; (%))	2 (20)
Outborn (n; (%))	0
Apgar 1 min (median [IQR])	6 [3; 6]
Apgar 5 min (median [IQR])	7 [6;8]
antenatal steroids (n; (%))	5 (62.5)

1e. Other intracranial pathology and ICH (`other ICH`) (n=8)

Gestational age median (range in weeks $^{+days}$)	33^{+6} (24^{+0} - 40^{+3})
Birth weight in g (mean and range)	2060 (650-3480)
Male (n; (%))	4 (50)
multiple pregnancy (n; (%))	2 (25)
Outborn (n; (%))	2 (25)
Apgar 1 min (median [IQR])	3 [1;5]
Apgar 5 min (median [IQR])	7 [4;9]
antenatal steroids (n; (%))	1 (12.5)
diagnoses	• haemorrhage and congenital hydrocephalus,<br>• traumatic grade III haemorrhage and subarachnoid haemorrhage,<br>• possible arterio-venous malformation and intraventricular haemorrhage<br>• intraparenchymal haemorrhage at near term<br>• congenital basal ganglia cysts and IVH<br>• possible neuronal migration disorder and IVH<br>• MCA stroke (two cases)

HIE – Hypoxic-ischaemic encephalopathy

ICH – Intracranial haemorrhage

IQR – interquartile range

IVH – Intraventricular haemorrhage

MCA – Middle Cerebral Artery

SD – standard deviation

Appendix Table 2: In-hospital deaths and physiological stability at time of last TLD. NB an additional three stable (category 3) infants died at home following discharge. Classification (Groups A-D) based on Verhagen et al. Physiological stability modified as detailed in Table 1 (main paper). NB an additional three stable (category 3) infants died at home following discharge

	**Number**	**Number of newborns and physiological stability**	
**Group A-** death during CPR	0		
**Group B-**withholding CPR-death on ventilator	1	Critically unstable (category 1)=1	P/IVH: n=1
**Group C-**extubation in moribund state to allow death in parents` arms	14	Critically unstable (category 1)=14	P/IVH: n= 3<br>HIE 3: n= 9<br>P/IVH+PVL=1<br>other ICH: n=1
**Group D-**elective extubation /non-reintubation	41	Stable/high level support (category 2)=8<br>Stable (category 3) =33	P/IVH: n= 21<br>HIE 3: n=13<br>P/IVH+PVL: n=4<br>other ICH: n=3

CPR – Cardiopulmonary Resuscitation

HIE – Hypoxic-Ischaemic Encephalopathy

ICH – Intracranial haemorrhage

P/IVH – Periventricular/Intraventricular haemorrhage

PVL – Periventricular Leukomalacia

TLD – Treatment Limitation Discussion

Appendix Table 3

Diagnosis and outcome in infants surviving to discharge whose parents agreed to treatment limitation, compared with infants whose parents did not agree to limitation of treatment

	Limitation (n=8)	No Limitation (n=14)
Diagnosis		
HIE – stage II	1 (12.5%)	1 (7.1%)
HIE – stage III	5 (62.5%)	3 (21.4%)
P/IVH – grade 3	0	3 (21.4%)
P/IVH – grade 4	2 (25%)	5 (35.7%)
PVL	0	1 (7.1%)
Other ICH	0	1 (7.1%)
Outcome		
GOS 1	1 (12.5%)	3 (21.4%)
GOS 2	1 (12.5%)	3 (21.4%)
GOS 3	0	0
GOS 4	3 (37.5%)	5 (35.7%)
N/A	0	2 (14.3%)
Died	3 (37.5%)	1 (7.1%)

GOS – modified Glasgow Outcome Scale

HIE – Hypoxic-Ischaemic Encephalopathy

ICH – Intracranial haemorrhage

N/A – not available (lost to follow-up)

P/IVH – Periventricular/Intraventricular haemorrhage

PVL – Periventricular Leukomalacia

Appendix Table 4

Diagnosis and outcome in infants with HIE surviving to discharge who received or did not receive therapeutic hypothermia

	Hypothermia (n=6)	No Hypothermia (n=4)
Diagnosis		
HIE – stage II	1	1
HIE – stage III	5	3
Outcome		
GOS 1	1	0
GOS 2	0	0
GOS 3	0	0
GOS 4	3	5
Died	2	1

GOS – modified Glasgow Outcome Scale

HIE – Hypoxic-Ischaemic Encephalopathy

Appendix Table 5: Outcome of survivors following Treatment Limitation Discussions

Case no	Disease group and details	Parental decisions	Treatment withheld/withdrawn	Age at last follow-up<br>Longterm outcome
1	HIE 2 (not cooled)	Parental decision unclear	None	3 years +<br>Quadriplegic CP GMFCS 5<br>Seizures, Single words, gastrostomy
2	HIE 3 (cooled)	Parents opted for continuing intensive care	None	3 years +<br>Spastic quadriplegic CP GMFCS 5<br>Gastrostomy feeds; Seizures; no meaningful communication
3	HIE 3<br>(not cooled)	Parental decision: not documented	None	CP GMFCS 5;<br>microcephaly, seizures; cortical visual impairment; bilateral hearing loss; gastrostomy<br>Death in childhood
4	HIE 3<br>(cooled)	Parental decision to limit life sustaining treatment	Withhold CPR	3 years +<br>Choreo-athetoid CP, GMFCS 5, gastrostomy
5	HIE 2<br>(cooled)	Parental decision to limit life sustaining treatment	Withhold NGT feeds/ Withhold CPR; reintubation	3 years +,<br>Ambulant, no apparent disability
6	HIE 3<br>(cooled)	Parental decision to limit life-sustaining treatment	Withhold reintubation<br>Withhold `active resuscitation`	3 years +<br>Quadriplegic CP GMFCS 4; gastrostomy
7	HIE 3 –<br>(not cooled)	Parental decision to limit life-sustaining treatment	Withhold CPR	3 years +<br>CP GMFCS 5; epilepsy; bilateral hearing loss, gastrostomy
8	HIE 3<br>(not cooled)	Parental decision to limit life-sustaining treatment	Provide NGT feeds, withhold CPR,	Death - infancy
9	HIE 3<br>(cooled)	Parental decision to limit life sustaining treatment	Withhold reintubation<br>Withhold NGT feeds	Death in infancy
10	HIE 3<br>(cooled)	Parental decision unclear	None	Death in infancy

11	Other ICH	Parents opting for continuing intensive care	None	3 years +<br>CP GMFCS 1; Speech delay, dysarthria;<br>Normal school with support
12	Unilateral P/IVH IV	Parental decision to limit life sustaining treatment	Withhold CPR, not for further escalation of intensive care	3 years +<br>CP GMFCS 1-diplegic Mild intellectual disability;<br>Special class mainstream school
13	Unilateral P/IVH IV contralateral grade II haemorrhage	Parental decision to continue intensive care	None	3 years +<br>Normal
14	Unilateral P/IVH IV contralateral grade III P/IVH	Parental decision to continue intensive care	None	3 years +<br>Normal
15	Unilateral P/IVH IV	Parental decision to continue intensive care	None	3 years +<br>Normal
16	P/IVH grade III/IV post-haemorrhagic hydro-cephalus	Parental decision to limit life sustaining treatment	Withhold CPR,<br>Withhold reintubation	Death in infancy
17	P/IVH III/IV – VP shunt	parental decision unclear	None	Survived to discharge-no follow-up data
18	Unilateral P/IVH IV	Continuation of intensive care	None	3 years +<br>CP-GMFCS4 ventriculoperitoneal shunt; hemianopia<br>severe developmental delay, gastrostomy
19	Bilateral P/IVH III	Parental decision unclear	None	<3 years<br>mild disability; reduced vision ; speech/language delay;
20	Bilateral P/IVH III	Parental decision to continue intensive care	None	3 years +<br>mild developmental delay; seizures
21	Bilateral P/IVH 3 + posterior fossa hemorrhage	Parental decision to continue intensive care	None	Survived to discharge<br>No longterm follow-up

22	P/IVH 4<br>+PVL-	Parental decision to continue intensive care	None	3 years +<br>CP- GMFCS 4-5; visual impairment; no meaningful communication, gastrostomy

CP=cerebral palsy;

GMFCS=gross motor function classification system;

P/IVH: peri-/intraventricular haemorrhage;

PVL: periventricular leucomalacia;

NGT=nasogastric tube; CPR = Cardiopulmonary Resuscitation;

Appendix Figure 1: distribution of number TLDs in relation to number of newborns

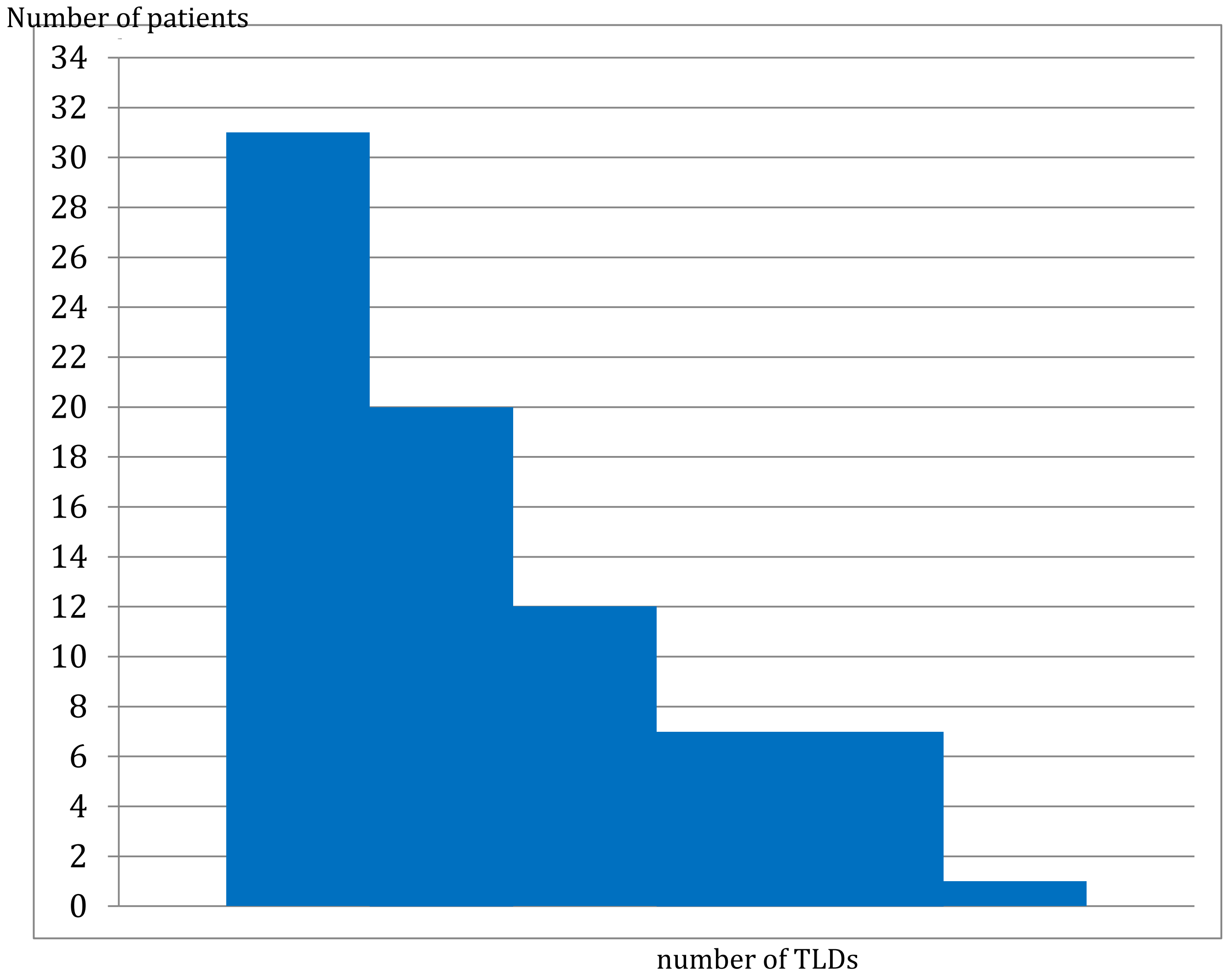

Appendix Figure 2 Stability at the time of treatment limitation discussion : 1=Critically unstable/moribund; 2=Stable but requiring high level of support; 3=Physiologically stable

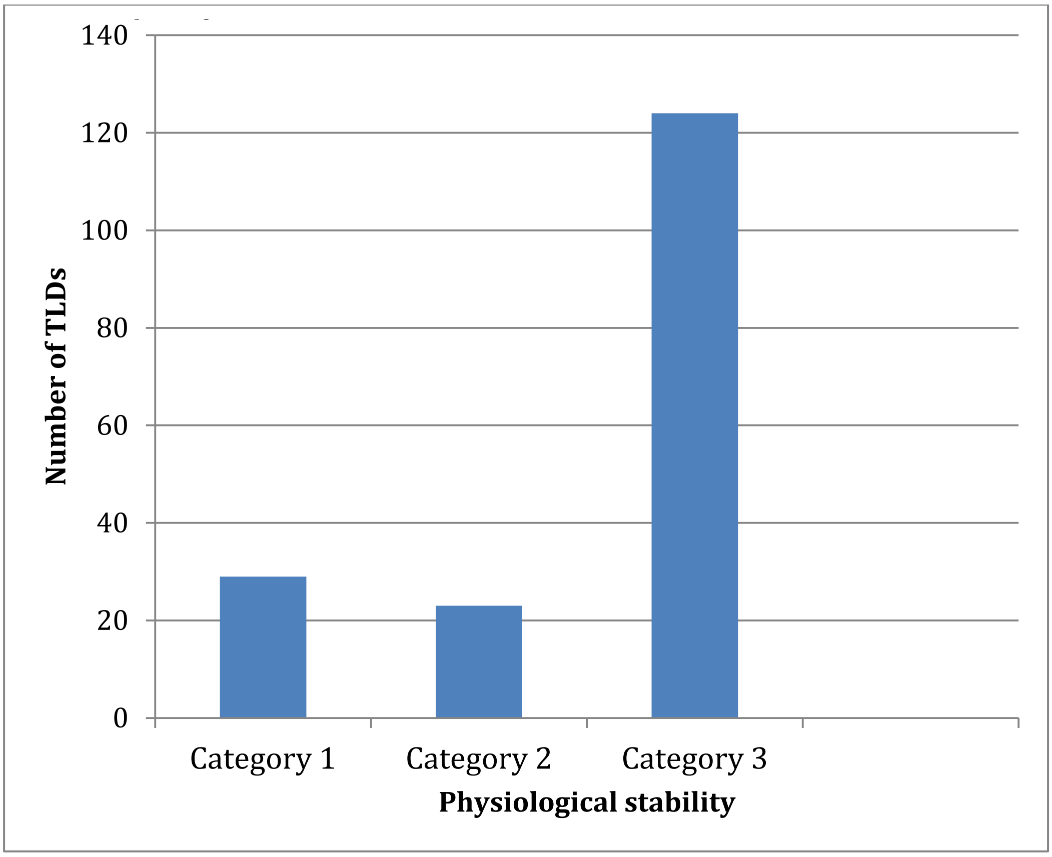

Supplement: Web supplement [file fetalneonatal-2014-307399-s1.pdf]
